# *In silico* analysis suggests differential response to bevacizumab and radiation combination therapy in newly diagnosed glioblastoma

**DOI:** 10.1098/rsif.2015.0388

**Published:** 2015-08-06

**Authors:** Andrea Hawkins-Daarud, Russell Rockne, David Corwin, Alexander R. A. Anderson, Paul Kinahan, Kristin R. Swanson

**Affiliations:** 1 Department of Neurological Surgery, Northwestern University, Chicago, IL 60611, USA; 2 Integrated Mathematical Oncology, Moffitt Cancer Center, Tampa, FL 33612, USA; 3 Department of Radiology, University of Washington, Seattle, WA 98195-7987, USA

**Keywords:** glioblastoma, hypoxia, bevacizumab, radiation, surgery, mathematical model

## Abstract

Recently, two phase III studies of bevacizumab, an anti-angiogenic, for newly diagnosed glioblastoma (GBM) patients were released. While they were unable to statistically significantly demonstrate that bevacizumab in combination with other therapies increases the overall survival of GBM patients, there remains a question of potential benefits for subpopulations of patients. We use a mathematical model of GBM growth to investigate differential benefits of combining surgical resection, radiation and bevacizumab across observed tumour growth kinetics. The differential hypoxic burden after gross total resection (GTR) was assessed along with the change in radiation cell kill from bevacizumab-induced tissue re-normalization when starting therapy for tumours at different diagnostic sizes. Depending on the tumour size at the time of treatment, our model predicted that GTR would remove a variable portion of the hypoxic burden ranging from 11% to 99.99%. Further, our model predicted that the combination of bevacizumab with radiation resulted in an additional cell kill ranging from 2.6×10^7^ to 1.1×10^10^ cells. By considering the outcomes given individual tumour kinetics, our results indicate that the subpopulation of patients who would receive the greatest benefit from bevacizumab and radiation combination therapy are those with large, aggressive tumours and who are not eligible for GTR.

## 1. Introduction

Glioblastoma multiforme (GBM) is the most aggressive form of primary brain tumour. The current standard of care involves surgical resection followed by concurrent radiotherapy and chemotherapy. Despite this aggressive treatment protocol, median survival remains about 14 months [1]. The Food and Drug Administration approved bevacizumab, an anti-angiogenic (AA) therapy targeting vascular endothelial growth factor A (VEGF A), in 2009 for recurrent GBMs based on promising imaging results. However, treatment results are spurious ranging from no notable response to significant imaging improvement often followed by an extremely diffuse pattern of tumour recurrence [2,3]. Recently, two separate phase III clinical trials, RTOG 0825 [4] and AVAglio [5], considered the role of bevacizumab in the upfront setting, as a concurrent treatment with chemotherapy and radiation following surgery. Both these trials found similar results in that there was a measureable increase in progression-free survival and no increase in overall survival. The RTOG 0825 study randomized 637 patients and the AVAglio study randomized 921 patients, but results did not account for possible subpopulations of patients that may have actually received a benefit in terms of progression-free survival or overall survival. That is, although the aggregate results of these phase III studies were disappointing for physicians and patients hopeful of a significant role for bevacizumab in tumour management, owing to the heterogeneity among patients both in terms of growth kinetics and hypoxic burden, there remains an open question of the potential benefits for identifiable subpopulations of patients. Our study focuses on the potential to identify individual patients for whom the addition of bevacizumab to surgical resection and radiation therapy may provide therapeutic benefits.

### 1.1. Anti-angiogenic therapy and hypoxia

Tumours often outgrow the native nutrient supply, resulting in regions of the tumour experiencing hypoxia or a lack of oxygen. A hallmark of cancer is that it is able to overcome this limitation and continue growing by inducing the angiogenic cascade [6]. AA therapy was initially conceptualized by Judah Folkman in the 1970s as an attempt to starve the tumour cells through decreasing the vasculature [7]. While angiogenesis is a complicated multistep process, the molecule VEGF A has been implicated as a key regulator of angiogenesis [8]. Many AA drugs, such as bevacizumab, are specifically designed to inhibit the action of VEGF A. However, GBM cells prefer to co-opt existing vasculature and recruit new vasculature only as a last resort [9]. As AA therapies tend to remove immature vessels and leave mature vessels intact, it is unclear whether GBM cells are ultimately exposed to hypoxia, or a lack of nutrients, as a result of therapy [10,11]. AA therapy at least transiently normalizes the vasculature, removing the immature and leaky vasculature while also repairing the blood–brain barrier. These findings raise the questions ‘is anti-angiogenic therapy actually re-oxygenating the tumour?’ and ‘if so, to what extent?’ The answers to these questions remain controversial. A small study of four human patients at recurrence indicated that bevacizumab concurrent with irinotecan did result in a reduction of hypoxia [12]. A recent additional case report also showed a decrease in hypoxia for a patient with recurrent anaplastic astrocytoma [13]. However, a study by Keunen *et al.* [14] in animals demonstrated an increase in hypoxia. As GBM tumours are heterogeneous, particularly in their hypoxic burden [15], it is likely that the answer to these questions will be patient-specific, making a generalized understanding of the effects of bevacizumab difficult to find with standard clinical trials where treatments are given in a one-size-fits-all fashion [16].

### 1.2. Measuring hypoxia

A primary reason why the state of tumour oxygenation during and post-AA therapy remains unclear is the difficulty in actually assessing levels of hypoxia. Only invasive techniques allow for direct measurement of the oxygen tension within the tissue, but even if measurements of relative hypoxia within the tumour are obtained, the spatial heterogeneity may result in the sampled regions not being adequate for defining the true hypoxic burden. To directly measure the spatial distribution of hypoxia *in vivo,* quantitative methods using positron emission tomography (PET) imaging with radiolabelled ligands that have been shown to co-localize with hypoxic regions, such as [F-18] fluoromisonidazole (FMISO), have been proposed [17]. While there are a few pre-clinical studies investigating how regions of high uptake on PET–FMISO correlate with biological characteristics relevant to radiotherapy [18] along with some theoretical studies investigating how radiation dose maps would change if PET–FMISO uptake was taken into consideration [19,20], it is not used clinically as it is not yet clear how outcomes would change based on this information [21].

The failure of the RTOG 0825 and AVAglio trials to demonstrate a survival benefit from the combination of radiation and bevacizumab can be considered surprising as the transient normalization of the vasculature within and around the tumour is hypothesized to increase the efficacy of both radiation and chemotherapy. Photon radiotherapy, the standard of care, depends on oxygen molecules for maximum DNA damage and it is thought that the normalized vasculature may re-oxygenate many regions of the tumour increasing overall efficacy [22]. Additionally, chemotherapeutic drugs are usually delivered through the vasculature system. The chaotic vessels in the tumour may be preventing the drug from being delivered, and thus normalization may increase the amount of drug delivered [23]. A driving hypothesis listed for the RTOG 0825 trial was that bevacizumab-induced normalization of vasculature may re-oxygenate the tumour increasing overall efficacy of both the ionizing radiation and alkalating agent chemotherapy [4]. For this reason, the work here focuses on the scenario of vascular normalization leading to tumour re-oxygenation and considers how this therapeutic effect would be realized for patients with different tumour kinetics.

By modelling similar therapies to those used in the two phase III clinical trials described above, this study aims to provide some initial hypothesis exploration with a mathematical model of GBM growth in the hopes of highlighting a potential subpopulation for whom bevacizumab may be able to provide an increased overall survival. We have developed a spatio-temporal biomathematical model for glioma proliferation and invasion incorporating the role of the angiogenic cascade. In a case study, this model was capable of reproducing a patient-specific PET image of hypoxia [24], and thus has demonstrated its potential for predictive modelling of hypoxia. By introducing an additional equation to capture fluid leakage from vasculature, we previously investigated the connection between what is measureable on T2-weighted magnetic resonance imaging (MRI) images and actual tumour extent post-treatment with AAs [25]. Recently, another group has also published work with a similar model specifically considering how invasion induced by bevacizumab, as an individual therapy, could also be explanatory of the different imaging responses and differences in progression-free survival [26]. The modelling effort here is focused on the complexities associated with modelling individual and combination therapies. In this paper, we extend our previous work by conducting a series of experiments to investigate patterns regarding the impact of AA therapy-induced hypoxic changes and the implications for synergistic activity with radiation therapy based on the tumour growth kinetics and size at the start of treatment. Additionally, we consider how gross total resection (GTR), by itself, is expected to reduce the hypoxic burden and the implications for combination therapy with AAs. This work ultimately demonstrates that different responses to these combination therapies may be explained by the spatial and temporal evolution of hypoxia within the tumour.

## 2. Material and methods

### 2.1. Mathematical model of glioma growth

The mathematical model is built around a simple model based solely on the proliferation and invasion rates of a tumour cell population, the proliferation invasion (PI) model [27–30]. To consider the impact of AA therapy on levels of hypoxia, we used a second model, based on this simple one, which incorporates the angiogenic cascade, the proliferation–invasion–hypoxia–necrosis–angiogenesis (PIHNA) model, first introduced in reference [31] and extended in [25].

In brief, the PIHNA model assumes the tumour is comprised of three different phenotypic tumour cells, normoxic (*c*), hypoxic (*h*) and necrotic (*n*). The vasculature cells (*v*) are also considered as a separate cell population as is the concentration of generic angiogenic factors (*a*). In words, it assumes the level of nutrients present in the local microenvironment, as inferred from the vessel density, determines whether the tumour cells exhibit normoxic or hypoxic phenotypes. That is, if there is a sufficient level of nutrients present, the cells will be well oxygenated, or normoxic, but if the nutrient level falls below a given threshold, the cells will become hypoxic. If the nutrients fall below an even lower threshold, the hypoxic cells will undergo necrosis. Normoxic tumour cells are allowed to move (invade) and divide while hypoxic cells are only allowed to move owing to restricted nutrients. Hypoxic cells produce a high concentration of angiogenic factors, which triggers an increase in the vessel density (i.e. angiogenesis).

For all the experiments in this paper, the model will be solved assuming spherical symmetry using MATLAB's pdepe function [32]. Thus, the solution is solved on a discretized domain composed of numerous grid cells at many time points. The reader is referred to the electronic supplementary material and [25,31] for further details, including the equations.

### 2.2. Comparing modelled tumour size to MRI

GBMs are generally monitored via MRI, specifically with contrast-enhancing T1-weighted (T1Gd) and T2-weighted scans. As we have done previously [28,30,31], we specify that the region predicted by our model to have a total cell density greater than or equal to 80% of the malignant cell carrying capacity (*K*) will correspond to the entire T1Gd abnormality and 16% will correspond to the T2 abnormality.

### 2.3. Modelling gross total resection

The definition of GTR is that the entire abnormality seen on T1-weighted post-contrast (T1Gd) MRI is removed. To define the impact of surgery from our model perspective, we assume the modelled T1Gd portion of the tumour is removed and consider the remaining tumour cells for the following analysis.

### 2.4. Modelling anti-angiogenic therapy

To model the effects of bevacizumab, we capture the two phenomena of inhibiting angiogenesis and normalizing pre-existing vasculature by requiring higher levels of angiogenic factor to be present to have the same level of ‘action’ in the contexts of both vessel proliferation and vessel permeability. Additionally, because the treatment is increasing the vessel efficiency, the level of vasculature needed for a cell to be normoxic will decrease, which we can capture by modifying the cell conversion rates from hypoxic to normoxic and from normoxic to hypoxic. Further details can be found in [25] and in the electronic supplementary material.

### 2.5. Modelling radiation therapy

There are many different models of radiation cell kill of varying levels of complexity [33]. Here, we will focus on a simple model for the fraction of cell kill, namely the linear-quadratic (LQ). The LQ model relates the dose *d* in units (Gy) delivered to the tissue in *n* fractions to the percentage of cells killed, *F*_CK_, using two parameters related to dose–response, *α* (Gy^−1^) and *β* (Gy^−2^), through the relationship 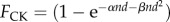. Consistent with references [30,34], we assume *α*/*β* = 10 Gy for early responding tumour tissue. The standard of care for delivering radiation to GBM patients involves delivering a total dose of 60 Gy delivered in fractions of 2 Gy, 5 days a week, for six weeks. While the concept of fractionation will be accounted for via the *n* in the definition of *F*_CK_, in this study, the cell kill is computed at a single time point, and whole-brain irradiation is assumed.

Rockne [30] studied the radiation sensitivity parameter *α* in a patient-specific context and found that it varied linearly with the parameter in the PI model for the net rate of proliferation, *ρ*. To capture the heterogeneity of radiation efficacy, we use this linear relationship when applying radiation for the individual simulations. It should be noted that this study did not explicitly separate cell kill owing to radiation and chemotherapy, thus the net radiosensitivity parameter (*α*) from Rockne [30] is also implicitly capturing the effects of chemotherapy.

It is known that photon radiation delivered to well-oxygenated tissue is more effective at killing cells than when delivered to poorly oxygenated tissue. This increase in efficacy is quantified through the oxygen enhancement ratio (OER) which has been seen to range between 1 and 3 [17,33]. In our model, this manifests as an inverse scaling modification to the parameters *α* and *β*, so that poorly oxygenated tissue would be associated with a scale factor reduction in radiation efficacy *α*_h_ = *α*/OER and *β*_h_ = *β*/OER. This is of particular interest in this paper as it is expected that AA therapy will reduce the hypoxic/poorly oxygenated portion of the tumour. Each grid cell will be associated with an effective OER, OER_e_, taken as the following weighted sum based on the relative hypoxia within that cell: 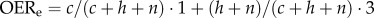, where *c, h* and *n* are the variables in the PIHNA model representing the normoxic, hypoxic and necrotic cell populations, respectively. We note that in regions entirely composed of normoxic cells OER_e_ = 1 and in regions entirely composed of hypoxic cells OER_e_ = 3, representing the most optimistic increase in radiation efficacy from re-normalization of the vasculature. Putting all these things together, for a given dose *d* delivered in *n* fractions, the radiation cell kill fraction is modelled as
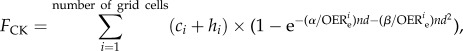
where *c_i_* and *h_i_* are the total number of normoxic and hypoxic cells in grid cell *i*, respectively, 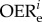 is the effective OER associated with grid cell *i*, the radiosensitivity parameter *α* will be calculated through the linear relationship with the pre-treatment net proliferation rate, *ρ*, determined in [30] and *α*/*β* = 10.

### 2.6. Tumour composition changes with timing of anti-angiogenic therapy

Patients were accepted to both RTOG 0825 and the AVAglio trial with minimal consideration of the size of their tumour. We hypothesize that the percentage of the tumour that is hypoxic will depend on the size of the tumour. To investigate the impact of varying tumour sizes at the start of AA treatment on hypoxic levels, we first chose parameter values that represented a patient with a very aggressive GBM (net invasion rate *D* = 305 mm^2^ yr^−1^ and net proliferation rate *ρ* = 83 1 yr^−1^). We then simulated tumour growth under three different scenarios of AA treatment: starting therapy when the tumour had reached 1.5, 2.5 or 3.5 cm as visible on T2/FLAIR MRI and continuing for 42 days, the typical length of a treatment session. For numerical details, see the electronic supplementary material. These starting sizes will be considered in all of the following simulations.

### 2.7. Experiment 1: exploring response across tumour kinetics and starting tumour size

As the range of radiographic response patterns seen clinically is broad, it should be expected that the re-oxygenation response would also be varied. Previous work has shown patient-specific values of proliferation and invasion range over several orders of magnitude [30,35]. To investigate how responses may be different for different underlying tumour growth kinetics, we simulated tumour growth in spherical symmetry under treatment for several combinations of proliferation rates, *ρ* (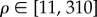 1 yr^−1^), and invasion rates, *D* (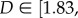
143] mm^2^ yr^−1^). The specific combinations that were run correspond to a Cartesian product of 11 *ρ*-values and 13 *D*-values spaced logarithmically within these ranges. The three tumour sizes, 1.5, 2.5 and 3.5 cm, were again considered as starting points for therapy to capture the heterogeneity in sizes at presentation seen clinically.

To capture the response in terms of a single number for each tumour, we used a metric of therapeutic re-oxygenation efficacy, *R*_1_ defined as the absolute change in the number of hypoxic cells between the two time points of just prior to AA therapy, *t*_1_ and 14 days into therapy, *t*_2_. The 14-day time point was chosen to correspond with the time of overlap of these therapies in the RTOG 0825 trial. Mathematically, this is written as
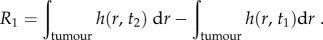


Here, *h*(*r*, *t_i_*) is representative of the number of hypoxic cells at location *r* and time *t_i_*.

### 2.8. Experiment 2: comparing with an untreated virtual control

A major benefit of using mathematical models to investigate questions such as those regarding treatment is that one can not only assess precisely what the difference is between the tumour pre-treatment and post-treatment, but one can also assess the difference between the tumour post-treatment and the tumour at the same time point without therapy. In essence, the model provides an untreated virtual control. Thus, experiment 2 is a two-armed version of experiment 1 where the two arms are tumours treated with AA therapy and tumours receiving no treatment. For this experiment, the response metric, *R*_2_, is defined as the difference in the total number of hypoxic cells seen in a tumour not having received therapy and the same tumour having received AA therapy for 14 days. Mathematically, this can be written as
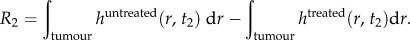


### 2.9. Experiment 3: evaluating efficacy of combination therapy with radiation

To more fully address the question of the efficacy of combination therapy involving AA and radiation therapy, we tested how many cells would be killed as a result of combination therapy both after two weeks of AA therapy, allowing some time for vascular renormalization, and the virtual control at the same time point. Specifically, the control arm did not have any AA therapy administered, but cell kill, computed as *F*_CK_, was assessed two weeks after the tumour reached the particular size of interest. For the treated arm, when the tumour reached the size of interest, AA therapy was started and continued for two weeks at the end of which the radiation cell kill was similarly computed as *F*_CK_.

### 2.10. Experiment 4: impact of gross total resection on hypoxic tumour cell burden

All previous results are working from the assumption that no GTR has been performed. However, for many patients, the reality is that they undergo GTR prior to additional therapies. It seems very likely that GTR would alter the number of hypoxic cells in the system by removing the central necrotic core. We therefore consider how GTR, when performed at different tumour sizes, would impact the potential efficacy of combining AA therapy with radiation as defined through the number of remaining hypoxic cells.

## 3. Results

### 3.1. Tumour composition changes with timing of anti-angiogenic therapy

**Figure 1.**
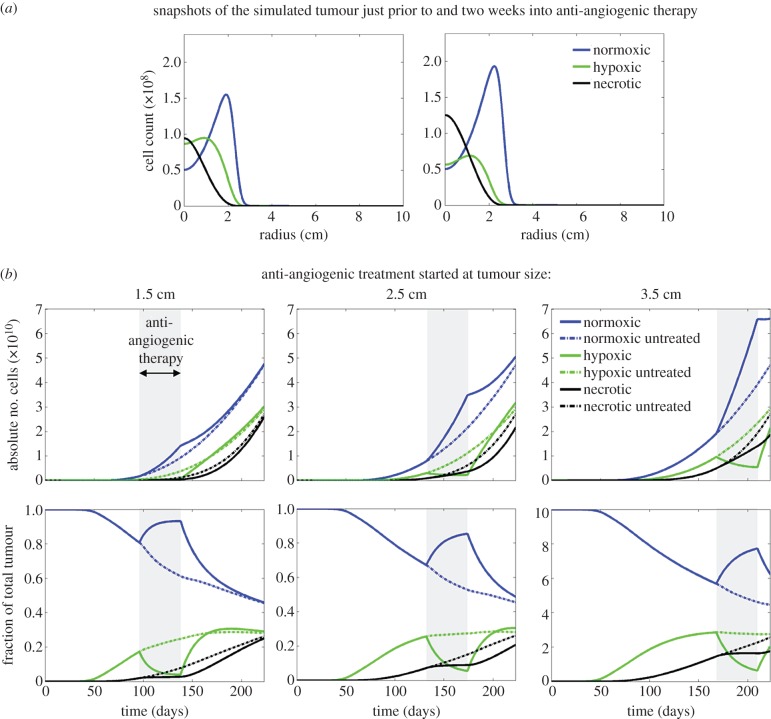
Simulation of an aggressive tumour (net invasion rate *D* = 305 mm^2^ yr^−1^ and net proliferation rate *ρ* = 83 1 yr^−1^) with anti-angiogenic therapy started at different time points in the tumour evolution. (*a*) Snapshots of the simulated tumour composition just prior to and after two weeks of anti-angiogenic therapy (treatment was started when the tumour size was 2.5 cm, the same as in the second column of *b*). One can see that the tumour is predicted to continue growing during therapy, however, the percentage of the tumour that is hypoxic is diminished under anti-angiogenic therapy. (*b*) For three different tumour sizes on T2-weighted MRI at the start of therapy, the top row shows the tumour composition as the total number of each phenotype of cell during the tumour progression, highlighting the time of treatment with the grey box. The second row presents the same data but as a percentage of the tumour cells being comprised of each phenotype. Solid lines are from treated simulations, whereas the dashed lines show the tumour dynamics if untreated for comparison. In all cases, the hypoxia is seen to diminish during therapy, however, the relative change depends on the size of the tumour.

Figure 1*a* shows two snapshots of the tumour simulation with treatment starting when the tumour's size was 2.5 cm. The first snapshot is of the tumour just prior to AA therapy, and the second snapshot is two weeks into therapy. The tumour continues to grow during treatment both in terms of radial outgrowth and in terms of higher total cell densities, suggesting no therapeutic effect. However, treatment does impact the relative proportions of the tumour populations, as the hypoxic population is at a higher density towards the centre of the tumour prior to treatment in contrast to post-treatment.

Figure 1*b* shows the evolution of the absolute number of cells in each population throughout the simulation (top row) and the fraction of the total tumour that the population composes. The solid lines show the results from the treated tumour, and the dashed lines show how these curves would have evolved without treatment. Generally, both rows show that AA therapy increases the number of normoxic cells compared with when left untreated and that the hypoxic and necrotic populations are reduced through therapy from where they would have been otherwise. The absolute deviation from the untreated curves, however, is more dramatic when treatment is started at later time points. Specifically, when treatment is started when the tumour size is 1.5 cm, the normoxic cell count within the tumour is predicted to be 1.4 × 10^10^ at the end of therapy versus 9.3 × 10^9^ for the corresponding time point of an untreated tumour. Similarly, for treatment started when the tumour radius is 2.5 cm, the normoxic cell count is 3.5 × 10^10^ at the end of therapy versus 2.2 × 10^10^ for an untreated tumour. Finally, if the treatment is started when the tumour size is 3.5 cm, the normoxic cell count is 6.6 × 10^10^ versus 3.9 × 10^10^. When thought of in terms of percentage of the tumour, however, one can see in figure 1*b* that if treatment is given when the tumour is small, the hypoxic population is minimized, and the vast majority of the tumour is normoxic and thus more susceptible to radiation. In contrast, when treatment is given to a larger tumour, even though the hypoxic percentage does decrease, the resulting normoxic percentage of the tumour is ultimately smaller as the necrotic population has begun to grow.

These simulations certainly support the hypothesis that AA treatment does re-oxygenate the tumour but that the benefit of combination therapy with radiation and chemotherapy is complicated to tease apart and will vary depending on the size of the tumour at the time of treatment.

### 3.2. Experiment 1: exploring response across tumour kinetics and starting tumour size

**Figure 2.**
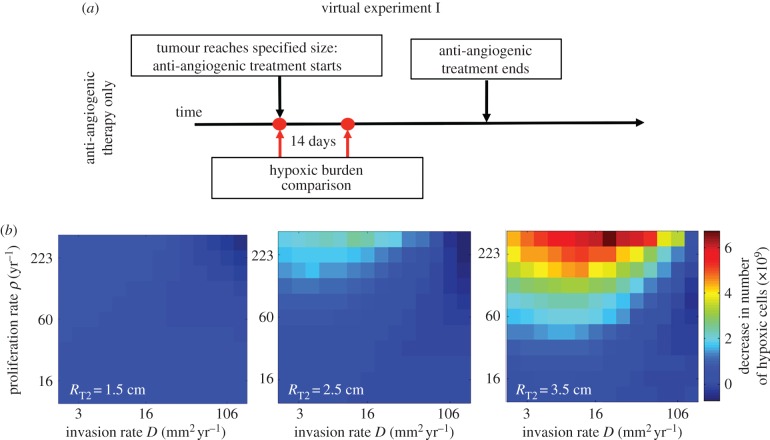
(*a*) Design of virtual experiment 1. (*b*) Absolute change in the number of hypoxic tumour cells after 14 days of anti-angiogenic therapy. The tumour size at the start of therapy is characterized by *R*_T2_, which is the radius presented on the T2-weighted MRI image. Each block of grid cells represents the full suite of growth kinetics tested with each grid cell representing a different ‘tumour’ defined by its net proliferation and net invasion rates.

The simulations illustrated in figure 1 only provide insights into the re-oxygenation response for one set of tumour growth kinetics. Figure 2 demonstrates the therapeutic response in terms of change in absolute hypoxic cell burden across a wide range of tumour kinetics. Each larger block represents a suite of simulations run with the individual sub-blocks representative of individual simulations run with specific rates of proliferation and invasion.

These results again demonstrate that the hypoxic percentage of the tumour is diminished under AA therapy. However, there are certain tumours that still have more hypoxia, in terms of absolute cell count two weeks into therapy than prior. This is particularly true for the smaller tumours with larger net rates of diffusion; when treatment is administered to larger tumours, it is still predicted that the slowly proliferating very diffuse tumours will ultimately have more hypoxic cells after two weeks of therapy. By looking across parameter space, we are also able to see that the re-normalization efficacy, defined by *R*_1_, is not uniform and thus would not be expected to be uniform across patients. While these results are suggestive of some patients receiving no benefit from re-normalization, they are insufficient to quantify how much ‘better off’ the patient is due to treatment. That is, these images only show the difference between the pre-treatment tumour and the two-weeks-into-treatment tumour. While this is the type of data available for comparison in the clinic, the question that should be of even more importance is the difference between the two-weeks-into-treatment tumour and the tumour if left untreated, the control.

### 3.3. Experiment 2: comparing with an untreated virtual control

**Figure 3.**
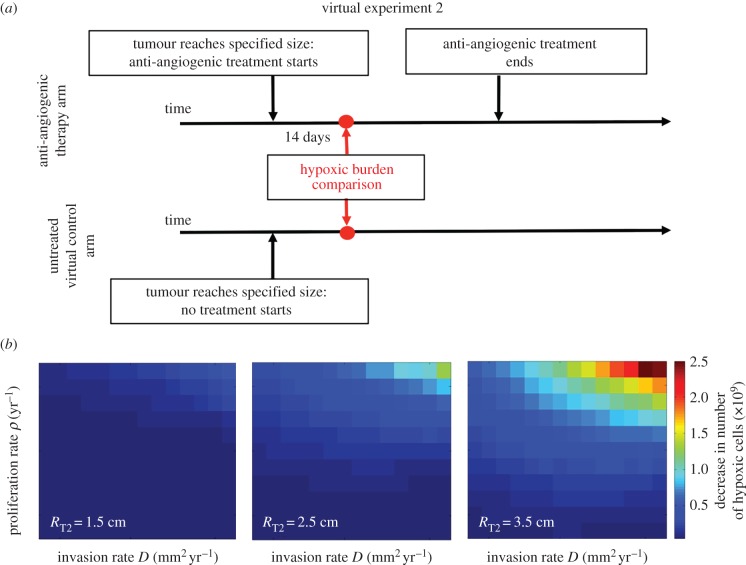
(*a*) Design of virtual experiment 2. (*b*) Comparison of the hypoxic burden between tumours two weeks into anti-angiogenic treatment and a virtual control having experienced no treatment. The largest benefit in terms of number of cells potentially salvaged from hypoxia and thus, increased radiosensitivity was observed in large tumours with high proliferation and high invasion rates.

Figure 3 shows similar blocks to those in figure 2, but they illustrate the difference, defined by *R*_2_, between the two-weeks-into-treatment tumour and its virtual control at the same time point, that is the tumour grown with the same parameters but never receiving treatment. Comparing figures 2 and 3, one can see the simulations that produced a negative difference for the two metrics (greater hypoxia post-therapy) were still receiving a benefit from therapy as there was a deviation in the amount of hypoxia two weeks into treatment when compared with if treatment had not happened at all. In addition, figure 3 suggests that the tumours with high proliferation and high invasion rates will experience the largest change in hypoxic burden owing to AA therapy.

### 3.4. Experiment 3: evaluating efficacy of combination therapy with radiation

**Figure 4.**
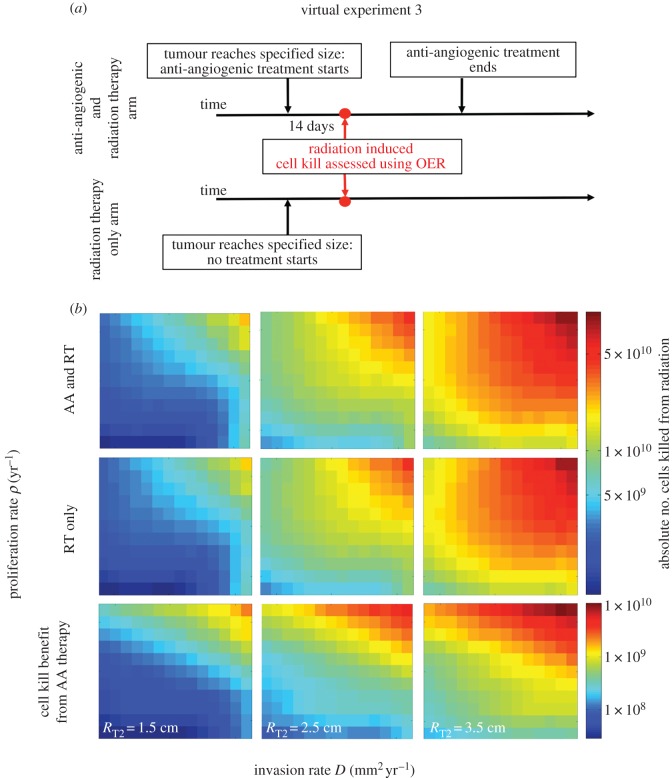
(*a*) Design of virtual experiment 3. (*b*) Radiation cell kill determined through the LQ model accounting for OER effects of hypoxic cells. Both colour bars in this figure are in terms of log base 10 to highlight the different orders of magnitude of cell kill. The third row highlights the additional cell kill due to combination therapy as the subtraction of the middle row from the top row, and thus has a different colour bar.

The top row of figure 4 shows the number of cells killed by radiation therapy after two weeks of AA therapy, the middle row shows the cells killed by radiation therapy at the same time point for a tumour having never received AA therapy, and the bottom row shows the difference between the two (radiation post-AA therapy minus the radiation only cell kill). While the combination therapy does always result in at least slightly more cells being killed, the bottom row highlights that not all tumours would be expected to receive the same benefit. Specifically, our model predicts that the combination of bevacizumab with radiation would result in an additional cell kill ranging from 2.6 × 10^7^ to 2.4 × 10^9^ cells for treatment starting when the tumour size is 1.5 cm, 8.5 × 10^7^ to 5.4 × 10^9^ cells for a tumour size 2.5 cm and 1.8 × 10^8^ to 1.1 × 10^10^ cells for a tumour size 3.5 cm. The general trend observed is that the highly proliferative and highly invasive tumours appear to have the most additional cells being killed, however, as the tumours get larger in size, the range of tumour kinetics receiving an appreciable change also increases.

### 3.5. Experiment 4: impact of gross total resection on hypoxic tumour cell burden

**Figure 5.**
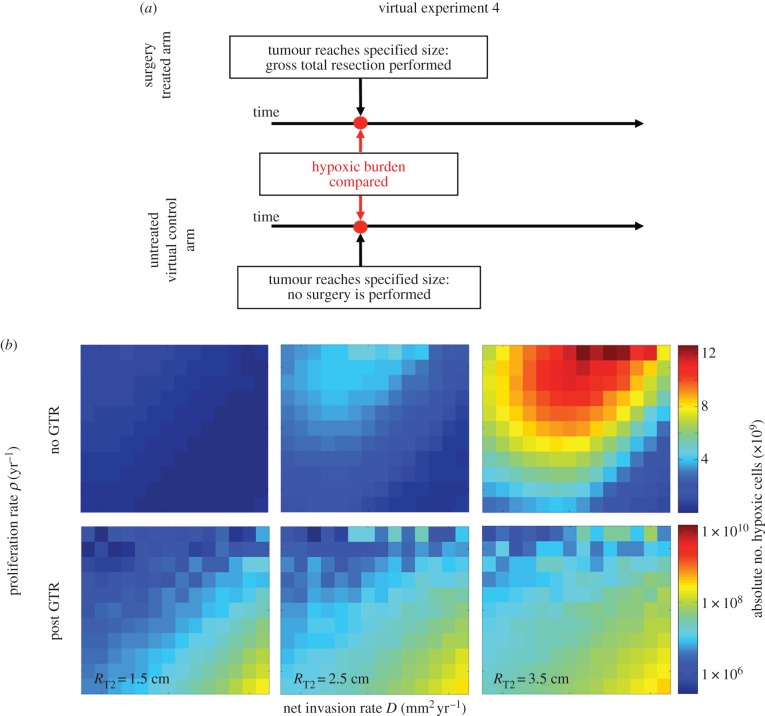
(*a*) Design of virtual experiment 4. (*b*) Comparison between hypoxic cell burden with or without gross total resection.

Results are illustrated in figure 5. As in the previous figures, the columns represent different possible sizes of the tumour at the time of resection, but here the top row shows the total number of hypoxic cells at that size just prior to GTR and the bottom row shows the hypoxic cells remaining immediately following GTR. These results show that for all tumour kinetics GTR removes the vast majority of the hypoxic cells, reducing the potential efficacy of the combination therapy. In fact, our model predicts that GTR would result in a removal of the hypoxic burden ranging from (11–99.97%), (35–99.99%) and (60–99.99%) for the surgery occurring when the tumour radius is 1.5, 2.5 or 3.5 cm, respectively. This is in agreement with previous work showing that most of the hypoxic region of GBMs is within the T1Gd abnormality [36]. Even so, the bottom row shows that there is a subpopulation with a higher number of hypoxic cells post-GTR which may receive more benefit than the others post-GTR from combination therapy, namely the slowly proliferating more diffuse tumours.

## 4. Discussion

Building on our previous work with our biomathematical model, this work aims to address how AA therapy may impact levels of hypoxia and the implications this has for combination therapy for different tumour growth and treatment response kinetics. Previously, we showed that by holding the modelling effects of treatment constant and only varying *D* and *ρ*, a wide range of image responses, analogous to what is seen clinically, were possible [25]. Here, we take this result one step further and show that while our model supports some level of re-oxygenation for all tested cases, the potential therapeutic benefit for combination therapies may vary across tumour kinetics.

While the parameters in our model regarding the effects of AA therapy have been estimated from literature and not rigorously fit to data, we believe it represents a useful framework to begin exploring such questions as how the net effects of AA therapy would re-normalize tumours given their expected hypoxic burden. Our work here is based on the assumption that AA therapy increases the vascular efficiency. We recognize that a possible limitation to our findings is that we consider only vascular normalization as there have been some reports of AA therapy increasing hypoxia in animal models [14]. This hypothesis was considered exclusively, however, as it was listed in the rationale for the RTOG 0825 study [4] and does have some credence given published studies on small patient cohorts [12,13]. It is true that this assumption results in all the virtual patients ultimately experiencing less hypoxia post-AA therapy in comparison with the situation if no therapy had been delivered, as demonstrated in figure 2; however, the degree of deviation varies dramatically between the patients. We want to emphasize that the value of this manuscript lies in the fact that clinical trials contain a mixed bag of seemingly similar patients. This paper merges our simplified understanding of the therapies being tested to a virtual cohort of patients to see how different distributions of these ‘similar’ patients may lead to different outcomes of the overall trial. While more work needs to be done to discover the actual expected timeline for ‘transient normalization’ [11], this work represents the first exploration, to the best of our knowledge, of what the basic hypothesis that ‘normalization occurs’ could quantitatively mean in a heterogeneous, human tumour population for combination therapy with radiation.

What is clear from our results is that the re-oxygenation effect is different not only for tumours with different growth kinetics, but also for tumours with the same growth kinetics with treatment started at different sizes. We showed this in the context of comparing the levels of hypoxia pre-treatment and two weeks into treatment as well as when comparing the hypoxic levels in tumours two weeks into treatment and their virtual control. We further explored the potential efficacy of combining radiation with AA therapy by testing the change in cell kill owing to the change in the hypoxic population. We note the omission of chemotherapy in our models is a possible limitation to our analysis. However, the formula for the *α*'s used in the radiation model was taken from a paper where the effect of chemotherapy was implicitly incorporated into the estimate. Thus, while we are not modifying the effect of bevacizumab on the two therapies, radiation and chemotherapy, independently, we are still implicitly accounting for the total effect on cell kill. For this reason, we believe our results are still meaningful in answering questions related to the expected efficacy of such combined therapies. These analyses combined hint at possible explanations for the negative results of the AVAglio and RTOG 0825 phase III clinical trials.

A large percentage of the patients in both the RTOG 0825 and the AVAglio clinical trials underwent some level of debulking surgery. The RTOG 0825 reported that 63% of their patients on the bevacizumab arm and 59% on the placebo arm underwent GTR and the vast majority of the other patients underwent partial resection. Similarly, the AVAglio trial reported 41% of the patients on the bevacizumab arm and 42% on the placebo arm underwent GTR with the majority of the others undergoing partial resection. Our exploration of remaining hypoxic tissue after GTR, figure 5, highlighted that no matter the tumour kinetics, tumours that have undergone GTR are predicted to have very low levels of hypoxic tissue post-surgery. Further, when comparing the tumour kinetics in figure 4 that appear to have the greatest potential for therapeutic efficacy when combining radiation or surgery, there appears to be little overlap. Specifically, figure 4 points to fast-growing tumours (the top and top right corners of the grid) as having the greatest potential efficacy, whereas figure 5 points to slow-growing diffuse tumours (the bottom right corner). This mismatch may be a key reason the two studies did not yield statistically significant differences in overall survival between the standard-of-care (control) arm and the arm treated with AAs in combination with radiation and chemotherapy.

Similarly, smaller tumours and tumours with lower proliferation rates do not have significant hypoxic populations. Our results here indicate that for patients with these low hypoxic tumours, combination therapy of AAs and radiation would not result in a greater cell kill and would thus not likely lengthen these patients' overall survival.

While none of our simulation experiments here directly predict the overall survival expected from the various therapies, the combined results suggest some general guidelines when considering what class of patients would likely benefit. If GTR is performed, the combination therapy should be reserved for larger tumours only (2.5 cm or greater prior to surgery) with emphasis on the tumours with less proliferative more invasive tumour kinetics. This comes from the observation that small tumours and larger, more proliferative tumours would have had the vast majority of their hypoxic burden removed during surgery and would have only minimally increased radiation induced cell kill owing to the addition of bevacizumab. If no GTR is performed, combination therapy should still be reserved for larger tumours but this time with emphasis on the highly proliferative and invasive tumours.

In the coming years, we expect many retrospective analyses will be performed using the data from both the AVAglio and RTOG 0825 clinical trials looking for subgroups that benefit from the combination therapy. This *in silico* analysis highlights the importance of focusing on individual patients' tumour growth kinetics and tumour size at presentation. As the analysis will be retrospective, assessment of the patient's hypoxic burden will not be available, but our methods of assessing rates of proliferation and invasion can be done using standard pre-treatment MRI images and can act as a surrogate marker for the amount of hypoxia individual tumours are experiencing.

## 5. Conclusion

Results from simulation studies using a mathematical model of tumour growth provide a testable hypothesis that there is a subpopulation of patients who could benefit from combination therapy including bevacizumab and radiation, namely those with large, aggressive tumours, and who are not eligible for GTR. These results may explain why the AVAglio and RTOG 0825 studies did not find a significant increase in overall survival, as approximately 50% of the patients on these trials underwent GTR. If retrospective analysis of the AVAglio and RTOG 0825 trial data is performed using individual patient characteristics, the criteria developed here may allow a quantitative classification of patients with a significant increase in overall survival. With so few treatment options available to GBM patients, it is important that options not be disregarded when no benefit on a population level is observed if there may be a subgroup of patients that would receive benefit. We believe this study represents a first step in identifying such subgroups.

## Supplementary Material

Equations and Numerics
