# Supplementary material for: In silico analysis suggests differential response to bevacizumab and radiation combination therapy in newly diagnosed glioblastoma
Source: J R Soc Interface. 2015 Aug 6;12(109):20150388. doi: 10.1098/rsif.2015.0388 (PMC4535409; doi:10.1098/rsif.2015.0388)
Supplement: Equations and Numerics [file rsif20150388supp1.doc]

*In Silico* Analysis Suggests Differential Response to Bevacizumab and Radiation Combination Therapy in Newly Diagnosed Glioblastoma

**Supplemental Material**

This mathematical model considered in this paper assumes the tumor is comprised of three different phenotypic cells, normoxic (c), hypoxic (h), and necrotic (n). The vasculature cells (v) are also considered as a species as well as a generic population of angiogenic factors (a). In words, it assumes the level of nutrients present in the local microenvironment, as inferred from the number of vasculature cells, determines whether the present tumor cells will exhibit normoxic or hypoxic phenotypes. That is, if there is a sufficient level of nutrients present, the cells will remain normoxic, but if the nutrient level falls below a given threshold, the cells will become hypoxic. If the nutrients fall below an even lower threshold value, the hypoxic cells will undergo necrosis and remain in the necrotic cell population. Normoxic tumor cells are allowed to move (invade) and divide while, due to restricted amounts of nutrients, the hypoxic cells are only allowed to move. The hypoxic cells produce a large amount of angiogenic factors which ultimately cause an increase in the number of vasculature cells. These angiogenic factors additionally cause the vasculature to become leaky, which in turn allows edematous fluid to escape the vasculature into the extracellular space which diffuses and is slowly absorbed and removed from the system. Mathematically, it is written as follows:


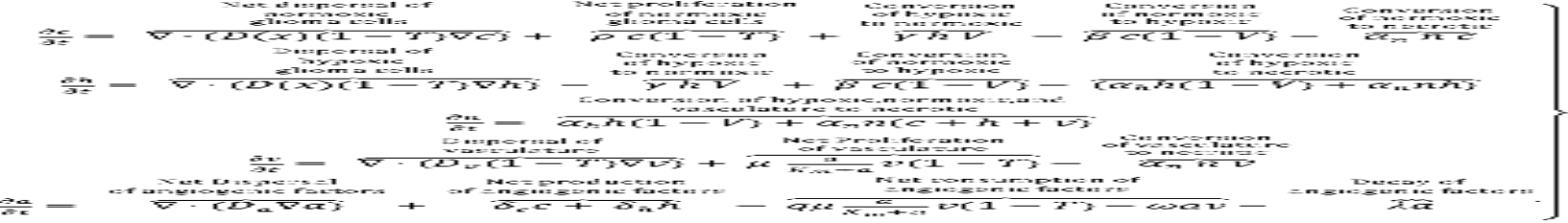
 (1)

In these equations,
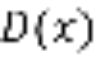
 is the net rate of invasion (mm^2^/yr) and is a piecewise constant, with nonzero values in the gray and white matter, Dg and Dw, respectively, with Dw>Dg, and zero in the regions of CSF. Additionally,
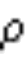
 (1/yr) is the net proliferation rate of the normoxic cells,
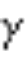
 (1/yr) and
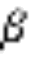
(1/yr) are the maximum conversion rates between the hypoxic and normoxic cell populations,
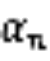
(1/yr) is the rate at which cells undergo necrosis when in contact with necrotic cells,
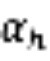
(1/yr) is the rate of conversion of hypoxic cells to necrotic cells when nutrient levels fall too low,
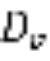
 (mm^2/^yr) is the rate of dispersal of vasculature cells, estimated from
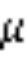
 (1/yr) is the vasculature proliferation rate, estimated from ,
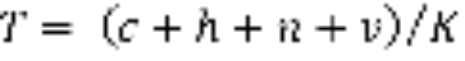
 (dimensionless), where
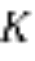
 is the carrying capacity (cells/mm^3^), and
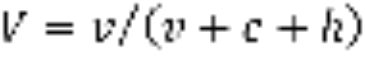
 (dimensionless) and is a surrogate for the local vasculature efficiency. The vasculature is assumed to proliferate according to a Michaelis-Menten type I form based on the interaction with the angiogenic factors where *K_m_* is the level of angiogenic factors required for the vasculature to reach half of the potential growth rate,
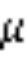
. Angiogenic factors are produced by both normoxic and hypoxic cells with rates
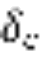
 (1/yr) and
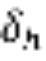
 (1/yr) respectively, with
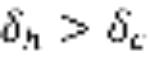
 and are consumed by the vasculature for both regular vasculature maintenance (with rate
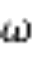
(1/yr)) and for vasculature proliferation (with rate
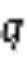
 (1/yr)). The angiogenic factors are assumed to decay over time with rate
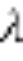
 (1/yr) and disperse with rate
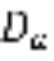
 (mm^2^/yr). Values for parameters related to the angiogenic factors were derived in part from work done in .

Homogeneous Neumann boundary conditions are assumed at the boundary to ensure no fluid or cells leave the brain through the skull. The reader is referred to for further details.

**Modeling Anti-Angiogenic Therapy** Bevacizumab is a drug specifically targeted at the molecule vascular endothelial growth factor A (VEGF A) which stimulates new vessel growth by binding with the vascular endothelial growth factor receptor (VEGFR2) on endothelial cells. Bevacizumab attempts to inhibit angiogenesis by binding to the free molecules of VEGF A and preventing their interaction with VEGFR2. In tumorous areas, an unintended consequence of this drug is that beyond preventing the growth of new vessels, it also “normalizes” pre-existing vasculature . That is, once the stimulating angiogenic factors are reduced, vessels repair their leakiness and return to a normal size - making them more efficient nutrient deliverers.

These actions of treatment are approximated in our model by decreasing the parameter for conversion from normoxic to hypoxic (β) by a factor of 10, increasing the parameter for conversion from hypoxic and normoxic (γ) by a factor of 10, and increasing the required levels of angiogenic factors for inducing vascular growth and vessel permeability by 2, i.e. doubling *K_m_*, as supported by the studies in .

**Numerics** For simplicity, we consider in all simulations here a three-dimensional spherically symmetric tumor. In all simulations, the domain is taken to be
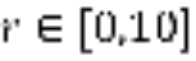
 for *r* the radius. The equations are spatially and temporally discretized on a grid satisfying the following three requirements depending on the proliferation and invasion rates being utilized:


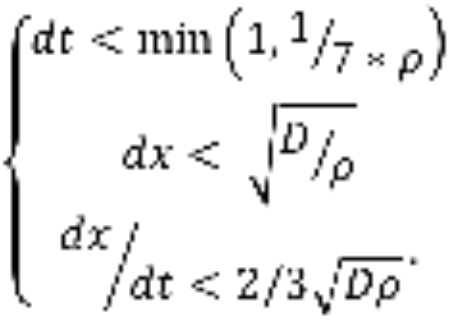


Matlab’s pdepe solver was used to solve the system until the tumor had reached a T1Gd size of 5 cm . The simulations were initiated with a small amount of normoxic cells distributed as


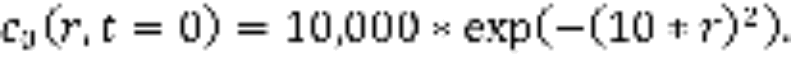


Vasculature is set at 3% of the cell carrying capacity throughout the domain based on estimates from , and all other quantities in the PIHNA-E model are initiated to zero. Unless otherwise stated, parameter values used in simulations are taken as specified in

**1. Sherratt, J.A. and J.D. Murray, *Models of Epidermal Wound-Healing.* Proceedings of the Royal Society B-Biological Sciences, 1990. 241(1300): p. 29-36.**

**2. Levine, H.A., et al., *Mathematical modeling of capillary formation and development in tumor angiogenesis: penetration into the stroma.* Bull Math Biol, 2001. 63(5): p. 801-63.**

**3. Xiu, M., et al., *Measurement of endothelial cell proliferation rate in vivo using 2H20 labeling: A kinetics biomaker of angiogenesis.* Faseb Journal, 2006. 20(4): p. A718-A718.**

**4. Mac Gabhann, F. and A.S. Popel, *Model of competitive binding of vascular endothelial growth factor and placental growth factor to VEGF receptors on endothelial cells.* Am J Physiol Heart Circ Physiol, 2004. 286(1): p. H153-64.**

**5. Serini, G., et al., *Modeling the early stages of vascular network assembly.* EMBO J, 2003. 22(8): p. 1771-9.**

**6. Swanson, K.R., et al., *Quantifying the role of angiogenesis in malignant progression of gliomas: in silico modeling integrates imaging and histology.* Cancer Research, 2011. 71(24): p. 7366-75.**

**7. Hawkins-Daarud, A., et al., *Modeling Tumor-Associated Edema in Gliomas during Anti-Angiogenic Therapy and Its Impact on Imageable Tumor.* Front Oncol, 2013. 3: p. 66.**

**8. Verhoeff, J.J.C., et al., *Concerns about anti-angiogenic treatment in patients with glioblastoma multiforme.* Bmc Cancer, 2009. 9: p. 444.**

**9. Jain, R.K., *Normalization of tumor vasculature: an emerging concept in antiangiogenic therapy.* Science, 2005. 307(5706): p. 58-62.**

**10. Desjardins, A., et al., *Evaluation of tumor response by dynamic contrast-enhanced magnetic resonance imaging in glioblastoma (gbm) patients treated with bevacizumab (bev) and irinotecan (CPT-11).* Neuro-Oncology, 2007. 9(4): p. 573-574.**

**11. Zhang, W., et al., *Acute Effects of Bevacizumab on Glioblastoma Vascularity Assessed with DCE-MRI and Relation to Patient Survival*, in *Intl. Soc. Mag. Reson. Med.* 2009.**

**12. MATLAB, *Release 2011b*. 2011, Natick, Massachusetts, United States: The MathWorks, Inc.**

**13. Blinkov, S.M.i. and I.i.a.I. Glezer, *The human brain in figures and tables; a quantitative handbook*. 1968, New York: Basic Books. xxxii, 482 p.**

**14. Swanson, K.R., et al., *Quantifying the role of angiogenesis in malignant progression of gliomas: in silico modeling integrates imaging and histology.* Cancer Res, 2011. 71(24): p. 7366-75.**
